# Bilateral Simultaneous Avulsion Fractures of the Proximal Tibia in a 14-Year-Old Athlete with Vitamin-D Deficiency

**DOI:** 10.1155/2015/783046

**Published:** 2015-09-06

**Authors:** Ziad Harb, Arfan Malhi

**Affiliations:** ^1^Croydon University Hospital, London Road, Surrey CR7 7YE, UK; ^2^Lewisham & Greenwich NHS Trust, Lewisham High Street, London SE13 6LH, UK

## Abstract

Fractures involving the proximal tibial epiphysis are rare and form 0.5% of all epiphyseal injuries. The specific anatomical and developmental features of the proximal tibial epiphysis make it vulnerable to unique patterns of fractures. Vitamin-D plays a vital role in bone homeostasis and its deficiency has an impact on fracture risk and healing. We present the first ever reported case of simultaneous bilateral proximal tibial physeal fractures in an athlete with vitamin-D deficiency. Treatment consisted of plaster immobilisation, and the patient made a full recovery and returned to preinjury level of activities. We report this case for its uniqueness and as an educational review of the importance of the developmental anatomy of the proximal tibia. We review the literature and discuss how the stages of the growing physis determine the type of fracture sustained.

## 1. Introduction

Fractures involving the proximal tibial epiphysis are rare and form 0.5% of all epiphyseal injuries [1]. These fractures are usually classified according to the Salter-Harris classification or by the system developed by Watson-Jones [2] and are later modified by Ryu and Debenham [3], specifically for the proximal tibial epiphysis. The age of the patient and mechanism of injury usually dictate the type of fracture sustained, and it is postulated that because of its specific anatomic arrangement, valgus or varus forces typically bypass the epiphysis and are transmitted directly into the metaphysis. Consequently, fractures of the epiphysis are extremely uncommon; however, tensile forces by the quadriceps acting on a flexed knee result in a flexion-type transitional fracture of the physis and these have been reported in the literature, referred to as either Salter-Harris II or Watson-Jones type IV fractures.

The role of vitamin-D in bone homeostasis is well-documented, and a deficiency has an impact on fracture risk and healing. We present a case of a 14-year-old athlete with vitamin-D deficiency, who sustained simultaneous bilateral proximal tibial physeal fractures.

## 2. Case Report

A fit and healthy 14-year-old male was admitted to our emergency department with bilateral severe knee pain following a jump during basketball. He described the pain starting on take-off and deteriorating upon landing, such that he was unable to weight-bear. The patient was a healthy, young man, participated in regular physical activity, and did not take any medications. He had a normal BMI and did not report any previous history of joint pain or trauma.

**Figure 1:**
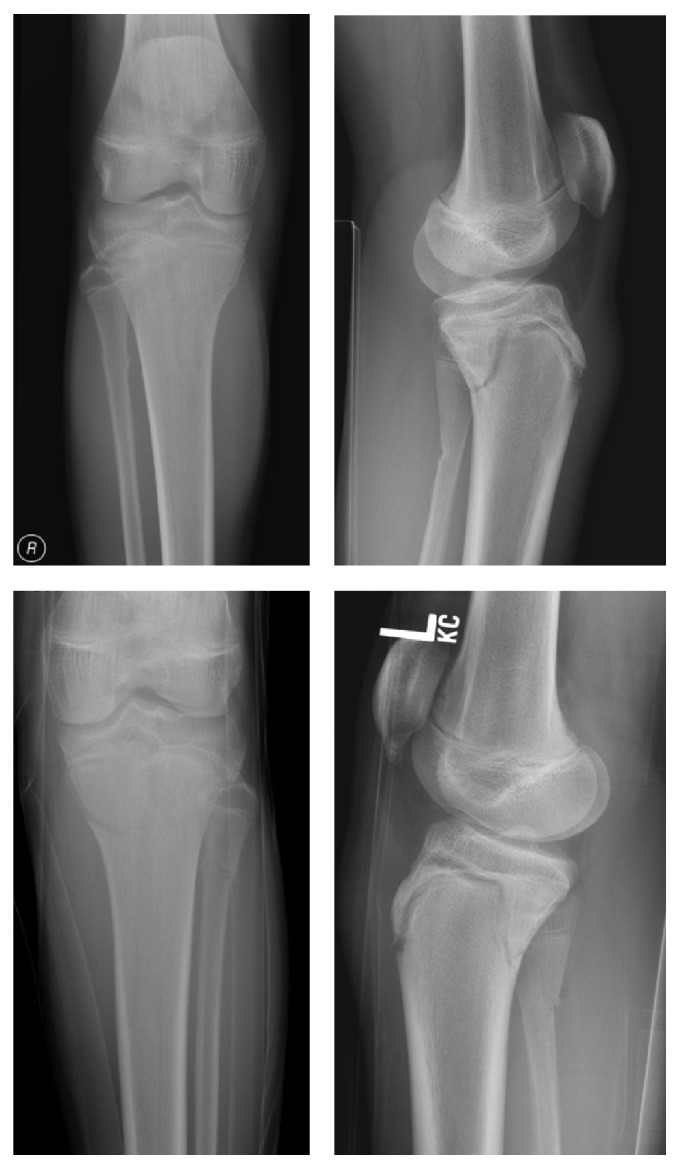
AP and lateral radiographs of both knees.

**Figure 2:**
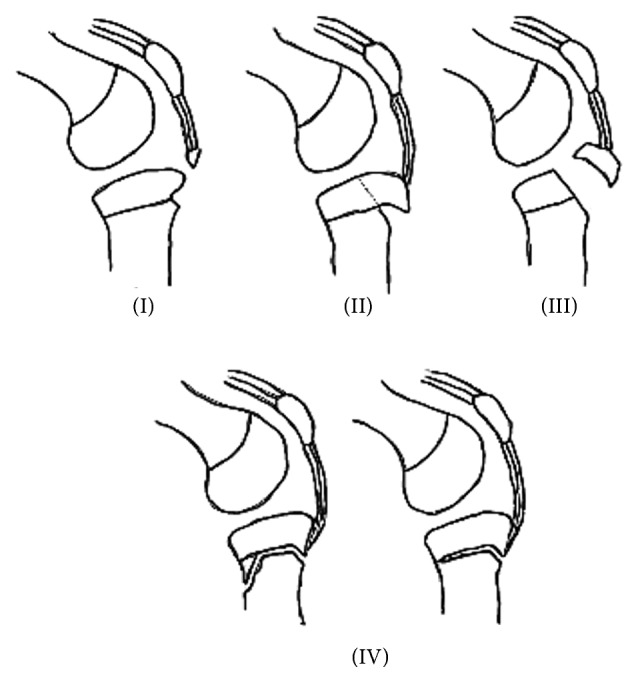
The modified Watson-Jones classification of tibial epiphyseal fractures. The original classification was for tibial tubercle fractures and included the first three types. Ryu and Debenham proposed type IV injury as an addition to the classification.

Clinical examination revealed bilateral knee effusions and tenderness anteriorly over the tuberosity and over the posteromedial aspect of the tibiae. The limbs were distally neurovascularly intact and there was no evidence of compartment syndrome. Movement was restricted due to severe pain; thus further examination was limited. Standard anteroposterior and lateral radiographs of the knees were obtained (Figure 1). These demonstrated bilateral undisplaced proximal tibial Salter-Harris type II fractures, which can also be classified as a type IV tibial tuberosity avulsion fracture according to the modified Watson-Jones classification (Figure 2). Laboratory investigations showed vitamin-D level of 20 nmol/L (normal value > 25 nmol/L, >10 ng/mL) and borderline low phosphate of 0.88 nmol/L (normal value 0.9–1.6 nmol/L). All other markers including calcium, liver function tests, and antibody screening were normal. A DEXA scan was performed and this showed normal bone density.

Nonoperative treatment was initiated with temporary casts, which were converted to full casts one week later. Treatment for vitamin-D deficiency was commenced. The patient remained non-weight-bearing for a total of six weeks, after which the casts were removed, clinical and radiological union was confirmed, and a gradual return to activities was instigated. At final follow-up six months following the injuries, the patient had returned to full activities and had a normal knee range of motion and no evidence of growth disturbance, leg length discrepancy, or angular malunion.

## 3. Discussion

Proximal tibial physeal injuries are rare, and it is thought that this is due to an inherent stability afforded by the anatomical arrangement of the epiphysis. The epiphysis has no medial or lateral attachments, with the medial collateral ligament being inserted directly onto the metaphysis; and on the lateral side structural support is offered by the fibula. Thus coronal plane forces bypass the epiphysis and are transmitted distally into the metaphysis. Sagittal forces, however, can cause significant epiphyseal trauma, which can be from either repetitive microtrauma such as the case in Osgood-Schlatter disease or acute fractures resulting from high-energy tensile forces created by a contracting quadriceps against a flexed knee. The fracture pattern is further determined by the stage of fusion of the epiphysis, which starts from the posterior aspect and propagates anteriorly until complete fusion at around the age of fifteen. A classification of proximal tibial epiphyseal injuries was proposed by Watson-Jones [2] and included three types of tuberosity fractures. This was modified by Ogden et al. [4] to include subtypes, and Ryu and Debenham subsequently added the “Watson-Jones type IV” fracture, described as a Salter-Harris type II of the proximal tibial epiphysis [3]. This classification system correlates with the stages of fusion of the proximal tibial epiphysis, such that the age of the patient at the time of injury, as well as the mechanism of trauma, will determine the type of fracture sustained. In our patient, the partial fusion of the proximal tibial epiphysis can clearly be seen and is reinforced by the fracture pattern which demonstrates that the energy imparted onto the proximal epiphysis passed through the open physis anteriorly and exited via a fracture line across the metaphysis posteriorly, resulting in a Salter-Harris type II of injury.

There are seven previous reports of bilateral simultaneous proximal tibial epiphyseal fractures in the literature [5–11], with only one case associated with an underlying diagnosis of osteogenesis imperfecta [11]; the others all occurred in healthy young adolescents. Our case is the first to report this injury in a patient with an underlying vitamin-D deficiency and serves to highlight the importance of having an index of suspicion for bone metabolic disorders in young patients with fractures, particularly in high risk groups such as those that have naturally dark skin, are obese, reside in areas with poor sun exposure, or have comorbidities such as Crohn's disease or epilepsy.

Treatment of these fractures is dependent on various factors, including the degree of displacement of the fragments. In our case, the displacement was deemed acceptable and the patient was successfully treated nonoperatively in plaster immobilisation. In the literature, there are reports of a variety of techniques of fixation, including plates and screws [10], cannulated screws [8], and tension band wiring [11]. Omar et al. [10] describe these injuries as “transitional fractures,” which should be differentiated from true Salter-Harris fractures because the tibia is reaching skeletal maturity and there is no concern about growth arrest and deformity; as such, they can be treated almost like adult proximal tibial fractures. Patients often have excellent outcomes whichever method they are treated by, and in the published cases there are no reported complications such as leg length discrepancy, angular malunion, or reduced range of motion. All the reported cases returned to preinjury level of sporting activity, although the follow-up in these patients is short and the long-term outcomes are not known.
